# Comparative Evaluation between Sulfasalazine Alone and in Combination with Herbal Medicine on DSS-Induced Ulcerative Colitis Mice

**DOI:** 10.1155/2017/6742652

**Published:** 2017-09-06

**Authors:** Mi-Rae Shin, Kyeong Jo Kim, Soo Hyun Kim, Su Ji Kim, Bu-Il Seo, Hyo-Jin An, Seong-Soo Roh

**Affiliations:** ^1^Department of Pharmacology, College of Korean Medicine, Sangji University, Wonju-si, Gangwon-do 26339, Republic of Korea; ^2^Department of Herbology, College of Korean Medicine, Daegu Haany University, 136 Shinchendong-ro, Suseong-gu, Daegu 42158, Republic of Korea

## Abstract

The present study aimed to investigate the comparative evaluation of pharmacological efficacy between sulfasalazine alone and sulfasalazine in combination with herbal medicine on dextran sodium sulfate- (DSS-) induced UC in mice. Balb/c mice received 5% DSS in drinking water for 7 days to induce colitis. Animals were divided into five groups (*n* = 9): Group I (normal group), Group II (DSS control group), Group III (DSS + sulfasalazine (30 mg/kg)), Group IV (DSS + sulfasalazine (60 mg/kg)), and Group V (DSS + sulfasalazine (30 mg/kg) + Cinnamomi Cortex and Bupleuri Radix mixture (30 mg/kg) (SCB)). Colonic pathological changes were analyzed using hematoxyline/eosin staining. The antioxidant, inflammatory, and apoptotic protein levels were determined using western blotting. SCB supplementation, as well as sulfasalazine, suppressed colonic length and mucosal inflammatory infiltration. In addition, SCB treatment significantly reduced the expression of proinflammatory signaling molecules through suppression of both mitogen-activated protein kinases (MAPK) and nuclear factor-kappa B (NF-*κ*B) signaling pathways and prevented the apoptosis of the colon. Moreover, SCB administration significantly led to the upregulation of antioxidant enzymes including SOD and catalase. Taken together, SCB treatment might offer a better treatment for human UC than sulfasalazine alone or may be useful as an alternative therapeutic strategy against UC, without any evidence of side effects.

## 1. Introduction

Ulcerative colitis (UC), with high incidences worldwide, is commonly referred to as chronic inflammatory bowel disease (IBD) and is characterized by an uncontrolled inflammatory condition of the intestinal mucosa [1, 2]. The main symptoms of UC are abdominal pain, mucous and bloody diarrhea, weight loss, and anemia. Generally, recommended therapies for UC patients include anti-inflammatory drugs (corticosteroids, prednisolone, aminosalicylic acid, and sulfasalazine), immunosuppressants (thiopurines, azathioprine, 6-mercaptopurine, and 6-thioguanine), and antibiotics (metronidazole and ciprofloxacin) [3–5]. Herein, sulfasalazine, composed of 5-aminosalicylic acid and sulfapyridine, has been used as a standard of care in UC for decades; however, it generates excessive oxidative stress after high dosage and long-term intake, resulting in severe adverse symptoms, such as blood disorders, hepatotoxicity, hypospermia, and male infertility [6, 7]. Accordingly, a sole treatment of sulfasalazine is not entirely satisfactory. Therefore, the therapeutic strategy for UC needs to focus on replacing the current therapy; in addition, the new therapy has to act locally at the site of inflammation to maximize efficacy, to increase convenience, and to minimize side effects [8].

Cinnamomi Cortex has been widely used as a herbal medicine for treating cold related disorders such as common cold and influenza [9]. Besides, a variety of pharmacological effects have been suggested: anti-inflammatory action, antibacterial activity, antiplatelet aggregation, sedative effect, improvement of blood circulation, and inhibition of cancer metastasis [10, 11]. Bupleuri Radix has been previously and is currently used to treat many liver diseases such as chronic hepatic inflammation and viral hepatitis in Traditional Chinese Medicine [12, 13]. Moreover, a previous study reported that Bupleuri Radix has an inhibitory effect of hypercellularity in nephritis [14]. Taken together, we predicted that Cinnamomi Cortex with anti-inflammatory effect and Bupleuri Radix with hepatoprotective and renoprotective effects may exert potential therapeutic benefits in ulcerative colitis. So, the present study was conducted to evaluate the pharmacological effect of sulfasalazine alone and in combination with Cinnamomi Cortex and Bupleuri Radix mixture in experimentally induced ulcerative colitis in mice.

## 2. Materials and Methods

### 2.1. Materials

DSS (molecular weight: 36,000–50,000 Daltons) was purchased from MP Biologicals (Santa Ana, California, USA). Sulfasalazine (purity ≥ 98%) and phenylmethylsulfonyl fluoride (PMSF) were purchased from Sigma-Aldrich (St. Louis, MO, USA). The protease inhibitor mixture solution and ethylenediaminetetraacetic acid (EDTA) were purchased from Wako Pure Chemical Industries, Ltd. (Osaka, Japan). 2′,7′-Dichlorofluorescein diacetate (DCF-DA) was obtained from Molecular Probes (Eugene, OR, USA). The pierce bicinchoninic acid (BCA) protein assay kit was obtained from Thermo Fisher Scientific (Waltham, MA, USA). ECL Western Blotting Detection Reagents and pure nitrocellulose membranes were supplied by GE Healthcare (Chicago, IL, USA). Rabbit polyclonal antibodies against nuclear factor-kappa B p65 (NF-*κ*Bp65; 1 : 1,000, SC-372), p47^phox^ (1 : 1,000, SC-14015), Rac1 (1 : 1,000, SC-217), superoxide dismutase (SOD; 1 : 1,000, SC-11407), glutathione peroxidase-1/2 (GPx-1/2; 1 : 1,000, SC-30147), Bax (1 : 1,000, SC-7480), Bcl-2 (1 : 1,000, SC-7382), monocyte chemoattractant peptide-1 (MCP-1; 1 : 1,000, SC-28879), and intercellular adhesion molecule-1 (ICAM-1; 1 : 1,000, SC-1511-R); goat polyclonal antibodies against tumor necrosis factor-*α* (TNF-*α*; 1 : 1,000, SC-1351) and interleukin-1*β* (IL-1*β*; 1 : 1,000, SC-1252); and mouse monoclonal antibodies against phosphor-extracellular signal-regulated kinase 1/2 (p-ERK1/2; 1 : 1,000, SC-7383), phosphor-p38 (p-p38; 1 : 1,000, SC-7973), cyclooxygenase-2 (COX-2; 1 : 1,000, SC-19999), inducible nitric oxide synthase (iNOS; 1 : 1,000, SC-7271), histone (1 : 1,000, SC-8030), and *β*-actin (1 : 1,000, SC-4778) were purchased from Santa Cruz Biotechnology, Inc. (Santa Cruz, CA, USA). Polyclonal antibody against c-Fos (1 : 1,000, #4384) was obtained from Cell Signaling Technology, Inc. (MA, USA). Mouse monoclonal anti-Caspase-3 (1 : 1,000, 3004-100) was purchased from BioVision Inc. (Mountain View, CA, USA). Rabbit polyclonal anti-reduced nicotinamide adenine dinucleotide phosphate oxidase 4 (NOX4) was purchased from LifeSpan BioSciences (Seattle, WA, USA). Rabbit anti-goat (1 : 3,000, SC-2774), goat anti-rabbit (1 : 3,000, SC-2004), and goat anti-mouse (1 : 3,000, SC-2005) immunoglobulin G (IgG) horseradish peroxidase- (HRP-) conjugated secondary antibodies were acquired from Santa Cruz Biotechnology, Inc. (Santa Cruz, CA, USA). All other chemicals and reagents were purchased from Sigma-Aldrich (St. Louis, MO, USA).

### 2.2. Test Material

In this study, dried Bupleuri Radix (100 g) was extracted 10 times with water and boiled at 100°C for 2 h with distilled water. After filtration, the water extracts were evaporated using a rotary evaporator at 45°C. In addition, Cinnamomi Cortex was extracted with 50% EtOH.

### 2.3. Experimental Animals and Induction of Colitis

Animal experiments were carried out according to the “Guidelines for Animal Experimentation” approved by the Ethics Committee of the Daegu Haany University with certificate number DHU2017-044. Eight-week-old male Balb/c mice weighing 22–24 g were purchased from Orient (Gyeonggi-do, Korea). Mice were maintained under a 12-hour light/dark cycle and housed at a controlled temperature (24 ± 2°C) and humidity (about 60%). After adaptation (1 week), acute colitis was induced by oral administration of 5.0% (w/v) DSS dissolved in drinking water, for 7 days [15]. For each experiment, the mice were divided into 5 experimental groups and 36 colitic mice were arbitrarily allocated into 4 groups (*n* = 9/group):

- Normal group
- DSS control group
- Sulfasalazine 30 mg/kg treated group (30 sulfa)
- Sulfasalazine 60 mg/kg treated group (60 sulfa)
- Sulfasalazine 30 mg/kg + Cinnamomi Cortex and Bupleuri Radix mixture 30 mg/kg treated group (SCB)

Normal mice received drinking water without DSS throughout the entire experimental period. Sulfasalazine was used as a positive reference agent and it was given at 30 or 60 mg/kg/day. Moreover, Cinnamomi Cortex and Bupleuri Radix mixture 30 mg/kg are composed of Cinnamomi Cortex 25 mg/kg and Bupleuri Radix 5 mg/kg in 5 : 1 proportions. The two herbs mixed well in sulfasalazine 30 mg/kg using a homogenizer before drug treatment. The entire colon was removed immediately and examined for gross mucosal injury. The colon tissue was immediately frozen in liquid nitrogen and blood samples were collected by cardiac puncture from anesthetized mice. Subsequently, the esophagus and serum were kept at −80°C until analysis.

### 2.4. Measurement of ROS Level in the Serum

Serum ROS level was measured by employing the method of Ali et al. [16]. 25 mM DCFH-DA was added to the serum. After incubation for 30 min, the changes in fluorescence values were determined at an excitation wavelength of 486 nm and emission wavelength of 530 nm.

### 2.5. Preparation of Cytosol and Nuclear Fractions

Protein extraction was performed according to the method of Komatsu with minor modifications [17]. Colon tissues for cytosol fraction were homogenized with ice-cold lysis buffer A (250 mL) containing 10 mM HEPES (pH 7.8), 10 mM KCl, 2 mM MgCl_2_, 1 mM DTT, 0.1 mM EDTA, 0.1 mM PMSF, and 1,250 *μ*L protease inhibitor mixture solution. The homogenate was incubated at 4°C for 20 min. And then 10% NP-40 was added and mixed well. After centrifugation (13,400 ×g for 2 min at 4°C) using Eppendorf 5415R (Hamburg, Germany), the supernatant liquid (cytosol fraction) was separated in a new micro test tube. The left pellets were washed twice by buffer A and the supernatant was discarded. Next, the pellets were suspended with lysis buffer C (20 mL) containing 50 mM HEPES (pH 7.8), 50 mM KCl, 300 mM NaCl, 1 mM DTT, 0.1 mM EDTA, 0.1 mM PMSF, 1% (v/v) glycerol, and 100 *μ*L protease inhibitor mixture solution suspended and incubated at 4°C for 30 min. After centrifugation (13,400 ×g for 10 min at 4°C), the nuclear fraction was prepared to collect the supernatant. Both cytosol and nuclear fractions were kept at −80°C before the analysis.

### 2.6. Immunoblotting Analyses

For the estimation of c-Fos, NF-*κ*Bp65, and histone, 12 *μ*g of protein from each nuclear fraction was electrophoresed through 10% sodium dodecylsulfate polyacrylamide gel (SDS-PAGE). Separated proteins were transferred to a nitrocellulose membrane, blocked with 5% (w/v) skim milk solution for 1 h, and then incubated with primary antibodies (c-Fos, NF-*κ*Bp65, and histone) overnight at 4°C. After the blots were washed, they were incubated with anti-rabbit or anti-mouse IgG HRP-conjugated secondary antibody for 1 h at room temperature. In addition, 7.5 *μ*g protein of each cytosol fraction of NOX4, p47^phox^, Rac1, Bax, Bcl-2, Caspase-3, SOD, catalase, GPx-1/2, COX-2, iNOS, TNF-*α*, IL-1*β*, MCP-1, ICAM-1, and *β*-actin was electrophoresed through 8–12% SDS-PAGE. Each antigen-antibody complex was visualized using ECL Western Blotting Detection Reagents and detected by chemiluminescence with Sensi-Q 2000 Chemidoc (Lugen Sci Co., Ltd., Gyeonggi-do, Korea). Band densities were measured using ATTO Densitograph Software (ATTO Corporation, Tokyo, Japan) and quantified as the ratio to histone or *β*-actin. The protein levels of the groups are expressed relative to those of the normal mouse (represented as 1).

### 2.7. Hematoxylin and Eosin (H/E) Stain of Colon Tissue

For microscopic evaluation, intestine tissue was fixed in 10% neutral-buffered formalin and, after embedding in paraffin, cut into 2 *μ*m sections and stained using hematoxylin and eosin (H/E) for microscopic evaluation. The stained slices were subsequently observed under an optical microscope and analyzed using the i-Solution Lite software program (Innerview Co.).

### 2.8. Statistical Analysis

The data are expressed as the mean ± SEM. Significance was assessed by one-way analysis of variance (ANOVA) followed by Dunnett's multiple comparison test using SPSS version 22.0 software (SPSS Inc., Chicago, IL, USA). Values of *P* < 0.05 were considered significant.

## 3. Results and Discussions

**Figure 1:**
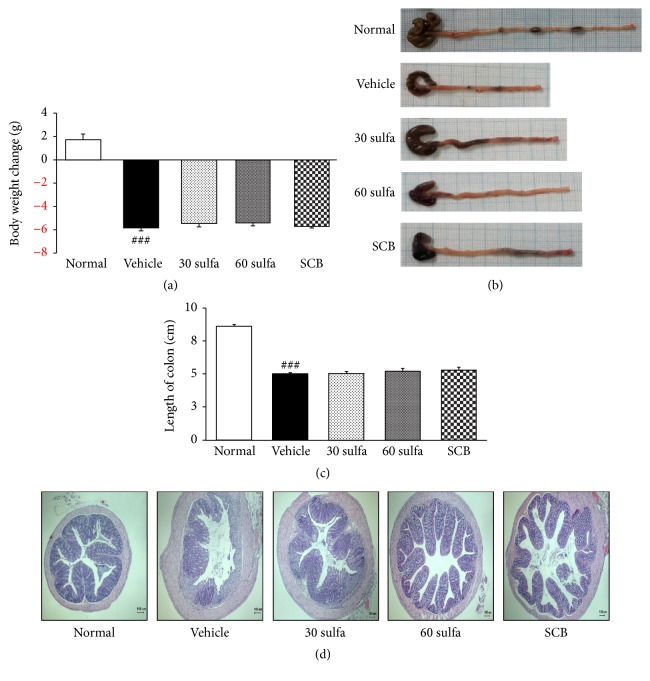
SCB alleviated dextran sodium sulfate-induced experimental colitis. (a) Body weight changes after induction of colitis by dextran sodium sulfate (DSS). (b) Macroscopic appearance. (c) Length of colon. (d) H/E staining of colon, magnification ×40. Data are mean ± SEM (*n* = 7). Significance: ^###^*P* < 0.001 versus normal mice.

Ulcerative colitis (UC), a chronic and complex autoimmune inflammatory disorder, is associated with a diverse dysfunction leading to overproduction of inflammatory cells and cytokine [18, 19]. In particular, the continuous progression of UC increases the risk of development of colorectal cancer (CRC) [20]. Accordingly, the development of a new complement remedy associated with concurrent use of sulfasalazine is needed for the effective alleviation of UC symptoms and the safety of long-term use because of the adverse effects of sulfasalazine [21]. The present study revealed, for the first time, the comparative evaluation of the pharmacological efficacy between sulfasalazine combined with Cinnamomi Cortex and Bupleuri Radix mixture (SCB) and sulfasalazine alone in a mouse model of UC. As shown in Figure 1, the DSS control group exhibited significantly increased body weight loss and decreased colon length (*P* < 0.001) in comparison with the normal group. Results in previous studies showed that the length of the colon was negatively correlated with the severity of experimental colitis [20, 22]. Sulfasalazine or SCB administration led to anti-inflammatory effects, including reduced body weight loss and less shortening of the colon length, whereas there was little significance in body weight change and colon length among the DSS-treated groups (Figures 1(a) and 1(c)). Colonic inflammation involves the disruption of the apparatus of colonic mucosa and ulceration, resulting in the infiltration of inflammatory cells such as inflammatory monocytes and macrophages and thickening of the lamina propria [23]. To investigate mucosal inflammation, we performed H/E staining. Colons in the normal group exhibited normal crypt morphology, abundant goblet cells, no signs of mucosal thickening, and complete absence of ulceration. On the contrary, microscopic damage was lower in SCB or sulfasalazine group than in the DSS control group (Figure 1(d)).

**Figure 2:**
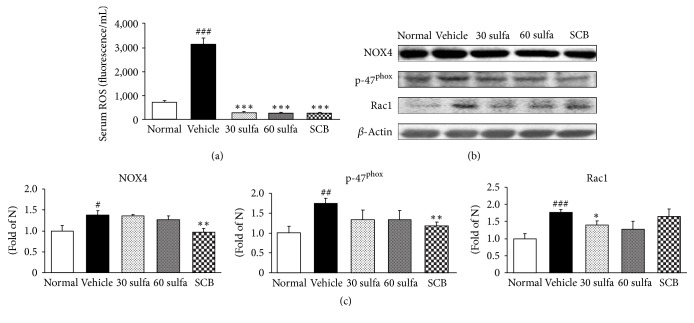
SCB decreased serum ROS and NADPH oxidase activity in the colon. (a) Serum ROS. (b) NOX4, p47^phox^, and Rac 1 protein expressions. Normal: normal mice. Vehicle: DSS control mice. 30 sulfa: sulfasalazine 30 mg/kg treated mice. 60 sulfa: sulfasalazine 60 mg/kg treated mice. SCB: 30 sulfa plus Cinnamomi Cortex and Bupleuri Radix mixture 30 mg/kg treated mice. Data are mean ± SEM (*n* = 7). Significance: ^#^*P* < 0.05, ^##^*P* < 0.01, and ^###^*P* < 0.001 versus normal mice and ^*∗*^*P* < 0.05, ^*∗∗*^*P* < 0.01, and ^*∗∗∗*^*P* < 0.001 versus DSS control mice.

**Figure 3:**
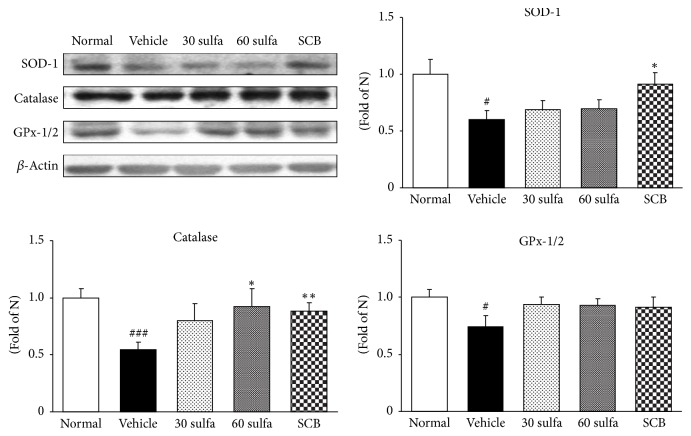
Effect of SCB on antioxidant proteins in DSS-induced colitis mice. SOD, catalase, and GPx-1/2 protein expressions. Normal: normal mice. Vehicle: DSS control mice. 30 sulfa: sulfasalazine 30 mg/kg treated mice. 60 sulfa: sulfasalazine 60 mg/kg treated mice. SCB: 30 sulfa plus Cinnamomi Cortex and Bupleuri Radix mixture 30 mg/kg treated mice. Data are mean ± SEM (*n* = 7). Significance: ^#^*P* < 0.05 and ^###^*P* < 0.001 versus normal mice and ^*∗*^*P* < 0.05 and ^*∗∗*^*P* < 0.01 versus DSS control mice.

Reactive oxygen species (ROS) are generated as part of the normal oxidative metabolism, yet cell damage is induced by their excess formation. Moreover, redox-active sulfur species, which are the widely known pathway of free radical generation by oxygen species, have been characterized as part of the sulfate assimilation pathway [24]. Since DSS is so highly sulfated, we estimate that it may lead to a sulfate load on cells and that this is associated with an elevation of the noticeable ROS, leading to acceleration of the inflammatory cascade. The reported clinical data show that ROS increases in colitis patients, causing oxidative cellular damage and promoting carcinogenesis [25, 26]. Previous studies indicated the importance of ROS-induced oxidative stress in the development of UC [22]. Besides, the key producers of ROS are NADPH oxidase enzymes including NOX4, p47^phox^, and Rac1 [27]. Overproduction of ROS via NADPH oxidase has been implicated in tissue damage observed in chronic inflammatory disorders [28] and plays vital roles in various biological activities, including host defense, cell growth and differentiation, stimulation of proinflammatory genes, and cell death [29]. In the present study, the DSS injury was markedly higher than in the normal group (*P* < 0.001), whereas treatment with SCB or sulfasalazine significantly decreased the damage by DSS (*P* < 0.001) (Figure 2(a)). The protein expressions of both NOX4 and p47^phox^, the markers of NADPH oxidase activity, in the colon were augmented in the DSS control group (versus normal group, *P* < 0.05 and *P* < 0.01, resp.). However, the SCB-treated group had significantly downregulated NADPH oxidase, whereas sulfasalazine-treated group exhibited a decrease without significance. Otherwise, Rac1 expression showed a tendency to decrease (Figure 2(b)). In general, ROS are known to be neutralized by endogenous antioxidant enzymes. SOD converts O^2−^ to H_2_O_2_, which is subsequently neutralized to water by catalase and GPx-1/2 [30]. The activity of enzymic antioxidants such as SOD, catalase, and GPx-1/2 was decreased in the DSS-induced group. Herein, SCB administration significantly increased the activity of SOD and catalase except for GPx-1/2 (without significance) (Figure 3). These findings indicated that SCB treatment of colitis may be reducing the extent of colonic injury by its antioxidant effect. Particularly, SCB supplementation was superior to sulfasalazine alone (Figure 3).

**Figure 4:**
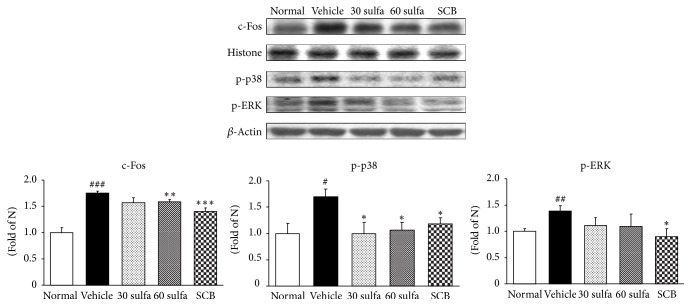
c-Fos, p-p38, and p-ERK protein expressions in DSS-induced colitis. Normal: normal mice. Vehicle: DSS control mice. 30 sulfa: sulfasalazine 30 mg/kg treated mice. 60 sulfa: sulfasalazine 60 mg/kg treated mice. SCB: 30 sulfa plus Cinnamomi Cortex and Bupleuri Radix mixture 30 mg/kg treated mice. Data are mean ± SEM (*n* = 7). Significance: ^#^*P* < 0.05, ^##^*P* < 0.01, and ^###^*P* < 0.001 versus normal mice and ^*∗*^*P* < 0.05, ^*∗∗*^*P* < 0.01, and ^*∗∗∗*^*P* < 0.001 versus DSS control mice.

**Figure 5:**
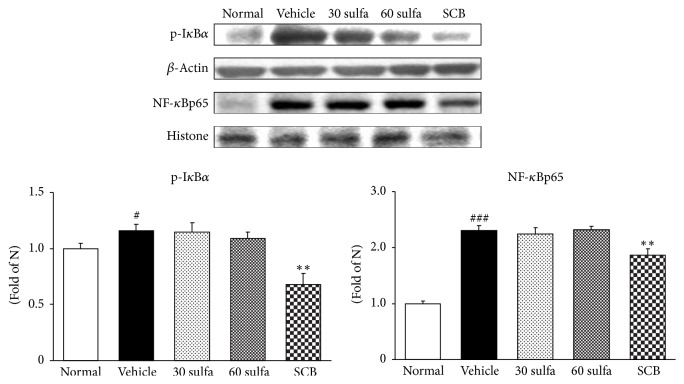
p-I*κ*B*α* and NF-*κ*Bp65 protein expressions in DSS-induced colitis. Normal: normal mice. Vehicle: DSS control mice. 30 sulfa: sulfasalazine 30 mg/kg treated mice. 60 sulfa: sulfasalazine 60 mg/kg treated mice. SCB: 30 sulfa plus Cinnamomi Cortex and Bupleuri Radix mixture 30 mg/kg treated mice. Data are mean ± SEM (*n* = 7). Significance: ^#^*P* < 0.05 and ^###^*P* < 0.001 versus normal mice and ^*∗∗*^*P* < 0.01 versus DSS control mice.

**Figure 6:**
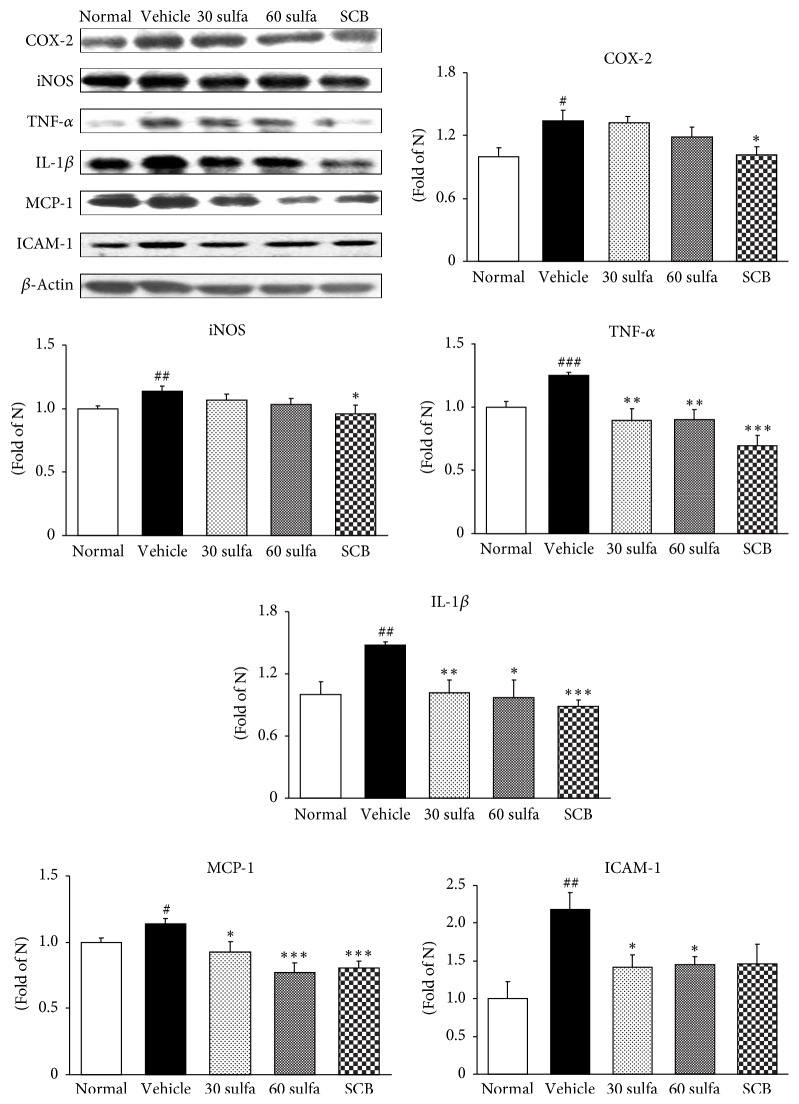
COX-2, iNOS, MCP-1, ICAM-1, TNF-*α*, and IL-1*β* protein expressions in DSS-induced colitis. Normal: normal mice. Vehicle: DSS control mice. 30 sulfa: sulfasalazine 30 mg/kg treated mice. 60 sulfa: sulfasalazine 60 mg/kg treated mice; SCB: 30 sulfa plus Cinnamomi Cortex and Bupleuri radix mixture 30 mg/kg treated mice. Data are mean ± SEM (*n* = 7). Significance: ^#^*P* < 0.05, ^##^*P* < 0.01, and ^###^*P* < 0.001 versus normal mice and ^*∗*^*P* < 0.05 and ^*∗∗∗*^*P* < 0.001 versus DSS control mice.

ROS overexpression activates MAPK including p38 and ERK1/2. The MAPK cascades on p38 and ERK1/2 have been proven to play major roles in the regulation of intracellular metabolism and gene or protein expression in many parts, including disease, apoptosis, and cellular responses to external stresses. Furthermore, the phosphorylation of p38 and ERK1/2 MAPK is also implicated by leading to the activation of nuclear transcription factors [31]. In this study, increased expressions of ERK1/2 and p38 were observed in the colons of the DSS control group (*P* < 0.05). As expected, SCB and sulfasalazine treatments were decreased via inhibition of their upstream c-Fos protein expression. Herein, SCB supplementation significantly attenuated activation of not ERK1/2 but p38 (*P* < 0.05) (Figure 4). As an important nuclear transcription factor, NF-*κ*B controls several important physiological processes, as well as immune and inflammatory responses. Prior to activation, NF-*κ*B is complexed with I*κ*B*α*, an inhibitory protein keeping NF-*κ*B in an inactive state in the cytoplasm. Induced by various stimuli, NF-*κ*B is released and translocates from the cytoplasm into the nucleus due to I*κ*B*α* phosphorylation, ubiquitinylation, and degradation [32]. Attempts to control mucosal inflammation through the use of agents that suppress the NF-*κ*B pathway have achieved some success in mouse models [33]. Similarly, SCB treatment has been shown to suppress the activation of NF-*κ*B by inhibition of I*κ*B*α* phosphorylation. Above all, SCB supplementation induced much lower activation of NF-*κ*B than sulfasalazine alone (*P* < 0.01) (Figure 5). And then NF-*κ*B participates in controlling the activation of various proinflammatory mediators such as inducible nitric oxide synthase (iNOS) and cyclooxygenase-2 (COX-2) and cytokines such as IL-1 and tumor necrosis factor-*α* (TNF-*α*), supporting a critical role in the pathogenesis of UC [34, 35]. As the result, the activation of NF-*κ*B results in the disruption of the oxidant/antioxidant balance [36]. TNF-*α* is crucial in recruiting immune cells at the sites of damaged tissues and in the pathogenesis of UC [37]. TNF-*α* and IL-1*β* as well as COX-2 and iNOS were noticeably amplified in the DSS control group. Our results also indicate that SCB significantly inhibits the induction of COX-2 and iNOS expressions and the production of proinflammatory cytokines such as TNF-*α* and IL-1*β*. These protein levels were downregulated to nearly normal levels (Figure 6). MCP-1 promotes monocyte infiltration into inflamed tissues and elevated levels of MCP-1 can be found in the intestinal mucosa of IBD patients [38]. Accordingly, reduced MCP-1 by SBC treatment might reduce the attraction of inflammatory cells into the intestine and thereby decrease inflammatory responses (Figure 6). Several studies have reported that TNF-*α* causes an increase in endothelial permeability and then leads to neutrophils recruitment to the gut in part through stimulating the synthesis of intracellular adhesion molecule (ICAM) [39, 40]. ICAM-1 is upregulated at sites of inflammation. Similar to advanced research, the DSS control group significantly increased compared with the normal group, whereas SCB treatment showed a tendency to decrease.

**Figure 7:**
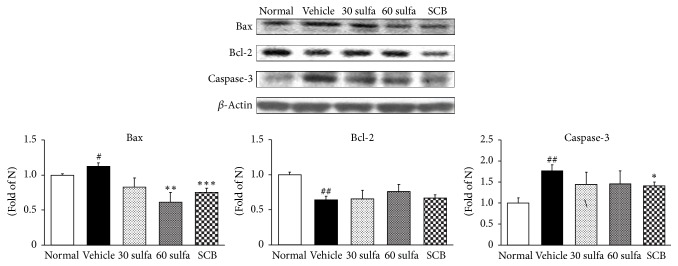
Bax, Bcl-2, and Caspase-3 protein expressions in DSS-induced colitis. Normal: normal mice. Vehicle: DSS control mice. 30 sulfa: sulfasalazine 30 mg/kg treated mice. 60 sulfa: sulfasalazine 60 mg/kg treated mice. SCB: 30 sulfa plus Cinnamomi Cortex and Bupleuri Radix mixture 30 mg/kg treated mice. Data are mean ± SEM (*n* = 7). Significance: ^#^*P* < 0.05 and ^##^*P* < 0.01 versus normal mice and ^*∗*^*P* < 0.05, ^*∗∗*^*P* < 0.01, and ^*∗∗∗*^*P* < 0.001 versus DSS control mice.

Apoptosis is considered to prevent excessive accumulation of nonfunctional cells in the tissue. Excessive exposure of intestinal mucosa to ROS under inflammatory conditions increases epithelial cell apoptosis [41], which is likely to change epithelial barrier integrity and contributes to intestinal damage. Bcl-2 is regarded as a prosurvival molecule, whereas Bax is a proapoptotic molecule as it binds to and antagonizes the effects of Bcl-2 [42]. Thus, Caspase-3 activation is an important event in cell death [43]. SCB showed substantial downregulation of proapoptotic genes, such as Bax and Caspase-3 (*P* < 0.001 and *P* < 0.05, resp.). Meanwhile, the Bcl-2 protein expression during UC did not show a marked difference as only a mild increase (Figure 7).

## 4. Conclusions

In conclusion, the present findings suggest that SCB is an effective inhibitor of DSS-induced colitis in mice. The administration of SCB to mice treated with DSS attenuated acute inflammation and apoptosis in the colon. Above all, SCB may exert a similar protective effect to sulfasalazine alone. Accordingly, SCB may be a promising herbal formula combined with sulfasalazine in the treatment of ulcerative colitis.
